# Glyphosate in Runoff Waters and in the Root-Zone: A Review

**DOI:** 10.3390/toxics3040462

**Published:** 2015-11-26

**Authors:** Lyndsay E. Saunders, Reza Pezeshki

**Affiliations:** Department of Biological Sciences, University of Memphis, 3700 Walker Avenue, Memphis, TN 38152, USA; E-Mail: pezeshki@memphis.edu

**Keywords:** root-zone exposure, non-target vegetation, vegetated buffer strips

## Abstract

Glyphosate is the most commonly-used herbicide in the world. The present review summarizes the discovery, prevalence, chemical and physical properties, mode of action and effects in plants, glyphosate resistance and the environmental fate of glyphosate. Numerous studies are reviewed that demonstrate that glyphosate may run off of fields where it is applied, while other studies provide evidence that plant roots can take up glyphosate. Non-target vegetation may be exposed to glyphosate in the root-zone, where it has the potential to remove aqueous glyphosate from the system. Further study on the effects of root-zone glyphosate on non-target vegetation is required to develop best management practices for land managers seeking to ameliorate the effects of root-zone glyphosate exposure.

## 1. Introduction

### 1.1. Background

Glyphosate was discovered as an herbicide in 1970 and became commercially available in 1974 as a post-emergent, non-selective herbicide [1]. Because it is a broad-spectrum herbicide, initial agricultural use of glyphosate was restricted to weed removal before planting with crops [2]. After its commercial introduction, glyphosate experienced commercial popularity as various formulations, such as Roundup^®^ (Creve Coeur, MO, USA). In 1996, genetic engineering led to the introduction of the first genetically-modified herbicide-resistant crop, Roundup Ready soybeans (*Glycine max*) [3]. The innovation of genetically-modified herbicide-resistance led to expanded use of glyphosate, making it the most applied herbicide globally.

### 1.2. Prevalence

Glyphosate is the most widely-used herbicide globally [2], although in recent years, its use has been restricted or outright banned in some countries. It is used most widely in agriculture, for field preparation and maintenance with herbicide-resistant crops. Non-agricultural uses include ornamental gardening and residential weed management, maintaining rights of way, forestry practices and ecological restoration [4].

Examining agricultural use statistics gives a sense of the extent of the use of glyphosate. The National Agricultural Statistics Service surveys in the United States selected states for different agricultural sectors to determine the amounts of agricultural chemicals used across the country, including glyphosate [5,6,7,8]. Table 1 summarizes the agricultural sectors in which glyphosate was used, the applied rates for the surveyed years and the percentage of planted acres receiving glyphosate. For soybeans, cotton, corn and nursery and floriculture crops, glyphosate was the most commonly-used herbicide [5,7,8]. For barley and sorghum, glyphosate was the second most commonly-used herbicide [6]. This is to be expected, because soybeans, cotton and corn all have genetically-modified herbicide-resistant varieties.

**Table 1:** Summary of glyphosate application in the United States for a given agricultural sector for a given year, as well as the percentage of hectares planted that received glyphosate [5,6,7,8].

Agricultural Sector	Amount Applied in Surveyed Year (kg)	% of Planted Hectares	Year Surveyed
Soybeans	45,530,000	89	2012
Corn	2,610,000	66	2010
Upland Cotton	4,811,000	68	2010
Sorghum	1,354,000	47	2011
Barley	428,000	35	2011
Nursery and Floriculture Crops	89,000	N/A	2009

N/A = not available.

### 1.3. Chemical and Physical Properties

Glyphosate is a phosphanoglycine compound [9]. The most commonly-applied form of glyphosate is in the form of its isopropylamine salt (IPA salt). Several chemical and physical characteristics for glyphosate are listed in Table 2. Commercial preparations of glyphosate contain three elements: IPA salt of glyphosate, a surfactant and water. The most commonly-used surfactant is polyethoxylated tallow amine (POEA), which promotes the penetration of glyphosate across the cuticle of target plants [4].

**Table 2:** Summary of the physical and chemical properties of glyphosate. Modified after Giesy *et al.* [4].

Common Name	Glyphosate
Synonyms	*N*-(Phosphonomethyl)glycine (acid)
	Glyphosate isopropylamine salt (IPA salt)
Chemical formula	C_3_H_8_NO_5_P (acid)
	C_3_H_9_N^.^C_3_H_8_NO_5_P (IPA salt)
Chemical Abstracts Service (CAS) No.	1071-83-6 (acid)
	38641-94-0 (IPA salt)
Molecular weight (g·mol^−1^)	169.09 (acid)
	227.2 (IPA salt)
Physical description	White crystalline powder
Melting point	200–230 °C
Boiling point	No data available
Water solubility	10,000–15,700 mg·L^−1^ at 25 °C
Vapor pressure	2.59 × 10^−5^ Pa at 25 °C
Octanol/water partition coefficient: log K_ow_	−4.59 to −1.70
Sorption partition coefficient: K_d_	3–1188; geometric mean (*n* = 28), 64
Sorption partition coefficient: K_oc_ (L·kg^−1^)	9–60,000; geometric mean (*n* = 28), 2.072
Acid dissociation constants	
pK_a1_ (first phosphonic)	0.8
pK_a2_ (carboxylate)	2.3
pK_a3_ (second phosphonic)	6.0
pK_a4_ (amine)	11.0

### 1.4. Plant Uptake, Transport and Metabolism

Glyphosate is applied directly to plant foliage through spraying [4] and enters the plant via diffusion [2]. The surfactant added to commercial preparations of glyphosate allows glyphosate to penetrate the plant cuticle by reducing the surface tension between the surface of the leaf and the sprayed droplet [4]. Once inside the plant, glyphosate enters the phloem and is transported to metabolic sinks via the symplastic pathway, which accounts for glyphosate’s property of being a systemic herbicide [1].

The physiochemical dynamics of symplastic glyphosate transport is explained by the intermediate permeability theory. This theory states that polar molecules, such as glyphosate, permeate membranes slowly and can enter phloem sieve tubes and be retained to allow for long-distance transport [10]. Glyphosate may also be transported within the plant xylem in the apoplastic pathway when taken up by roots [1]. For both foliar and root uptake, glyphosate translocation may be basipetal or acropetal, moving toward sink tissues, such as meristems, flowers and fruits [1,11,12,13,14]. Plants lack the ability to metabolize glyphosate [1]. Absorption of glyphosate through roots has been shown in several crop species, such as beets, barley, cotton, maize and rapeseed [13,15,16,17,18,19]. This exposure pathway is significant, because roots are the main intercept of glyphosate in field runoff.

### 1.5. Mode of Action and Effects in Plants

**Figure 1:**
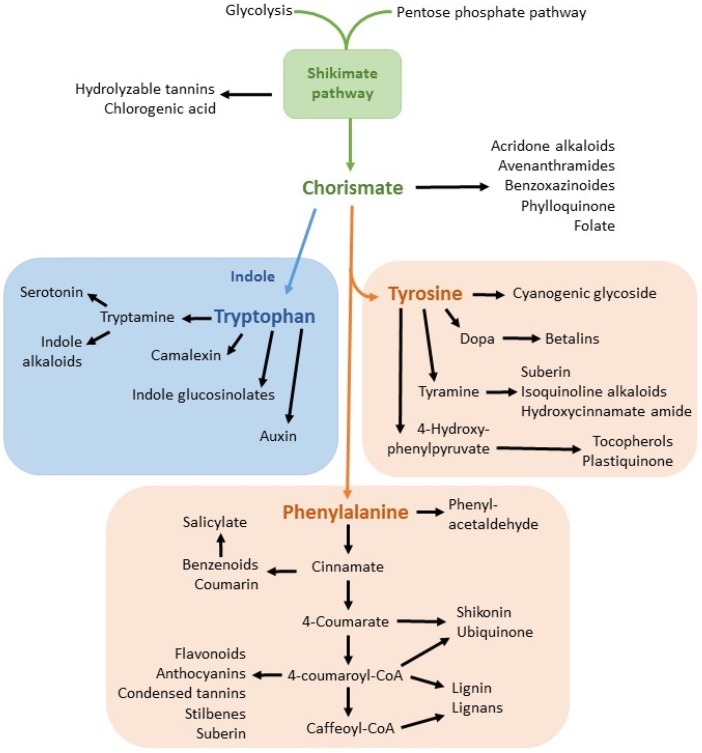
The shikimic acid pathway (shown in green) and selected metabolites. Chorismate is the common precursor molecule for the tryptophan pathway (blue) and the phenylalanine/tyrosine pathways (red). Modified after Maeda and Dudareva, [22].

Herbicides are classified based on their mode of action. Glyphosate is in the class of amino acid inhibitors [20]. Specifically, the synthesis of aromatic amino acids is disrupted due to the inhibition by glyphosate of enolpyruvylshikimic phosphate (EPSP) synthase [4,21]. This enzyme is essential to the shikimic acid pathway production of chorismate, an intermediate precursor molecule for the aromatic amino acids phenylalanine, tyrosine and tryptophan [4] and for a variety of secondary metabolites. The shikimic acid pathway and many of its metabolites are summarized in Figure 1.

Production of aromatic amino acids through the shikimic acid pathway is exclusive to plants, fungi and some microorganisms. This pathway is not present in higher animals, for whom amino acids must be consumed in the diet [23]. The lack of a shikimic acid pathway and, therefore, a lack of a target site may account for the apparent low toxicity of glyphosate in higher animals [4], although adverse effects of exposure have been documented [24].

In plants, the shikimic pathway takes place within the chloroplast [25]. An estimated 20% of assimilated carbon passes through this pathway [23]. Up to 35% of plant dry mass originates through this metabolic pathway [26].

The effects of glyphosate exposure develop several days after exposure [12,27,28,29]. Visually, symptoms of glyphosate exposure include foliar chlorosis followed by necrosis, leaf wrinkling and malformation and meristematic necrosis [25]. Physiologically, glyphosate exposure also results in reductions in photosynthesis and chlorophyll fluorescence [29,30,31,32,33,34] and in chlorophyll content [34,35,36,37]. Generally, these physiological effects decrease plant biomass production. However, glyphosate at low concentrations may induce hormesis, a stimulatory effect of some toxins at low levels [13,38,39,40,41,42].

### 1.6. Resistance to Glyphosate

Glyphosate resistance comes in two varieties: intentional and unintentional. Glyphosate resistance conferred through genetic engineering is intentional. Glyphosate resistance as an evolved trait due to high selection pressure from extensive glyphosate use is unintentional.

Glyphosate resistance in crops is conferred by the genetic engineering of an EPSP synthase gene from *Agrobacterium* sp. strain CP4 [43]. This gene produces an enzyme that is insensitive to glyphosate [44]. This technology has led to the introduction of six glyphosate-resistant crops in the following years: soybean (1996), canola (1996), cotton (1997), maize (1998), sugar beet (1999) and alfalfa (2005; removed from all commercial markets in 2007).

In 1996, the year of the introduction of genetically-engineered herbicide-resistant crop and 22 years after the commercial introduction of glyphosate, the first reports of glyphosate-resistant weeds began to surface in Australia. Today, some 225 confirmed cases of 29 glyphosate-resistant weed species exist globally, summarized in Table 3 [45]. Mechanisms of glyphosate resistance in weeds include two primary strategies: (1) a mutation that alters the target site for glyphosate (EPSP synthase) or that results in overexpression of EPSP synthase; or (2) changes in patterns of translocation and sequestration [43].

**Table 3:** Species, locations and year(s) of the discovery of glyphosate-resistant weed species across the world [45].

Family	Species	Locations	Year(s) Reported
Amaranthaceae	*Amaranthus palmeri*	AL, AR, AZ, DE, FL, GA, IN, IL, KS, KY, LA, MD, MI, MS, MO, NC, NM, OH, PA, SC, TN, TX, VA, United States	2005–2014
	*Amaranthus quitensis*	Argentina	2013
	*Amaranthus spinosus*	MS, United States	2012
	*Amaranthus tuberculatus*	IL, IN, IA, KS, KY, MN, MS, MO, NE, OH, OK, SD, TN, TX, United States	2005–2012
Asteraceae/Compositae	*Ambrosia artemisiifolia*	AL, AR, IN, KS, KY, MN, MS, MO, NE, NJ, NC, ND, OH, PA, SD, United States; ON, Canada	2004; 2006–2008; 2012–2014
	*Ambrosia trifida*	AR, IN, IA, KS, KY, MN, MS, MO, NE, OH, TN, WI, United States; ON, Canada	2004–2011
	*Bidens pilosa*	Mexico	2014
	*Conyza bonariensis*	NSW, QLD, SA, Australia; Brazil; Colombia; Greece; Israel; South Africa; Spain; Portugal; CA, United States	2003–2007; 2009–2011
	*Conyza canadensis*	AR, CA, DE, IN, IL, IA, KS, KY, MD, MI, MS, MO, NE, NJ, NC, OH, OK, PA, TN, VA, United States; Brazil; China; Czech Republic; Italy; Poland; Spain	2000–2003; 2005–2007; 2009–2013
	*Conyza sumatrensis*	Brazil; France; Greece; Spain	2009–2012
	*Parthenium hysterophorus*	Colombia	2004
Brassicaceae/Cruciferae	*Raphanus raphanistrum*	WA, Australia	2010
Chenopodiaceae	*Kochia scoparia*	AB, SK, Canada; CO, KS, NE, ND, OK, MT, SD, United States	2007; 2009; 2011–2013
Plantaginaceae	*Plantago lanceolata*	South Africa	2003
Poaceae/Gramineae	*Chloris elata*	Brazil	2014
	*Chloris truncata*	NSW, Australia	2010
	*Cynodon hirsutus*	Argentina	2008
	*Digitaria insularis*	Brazil; Paraguay	2005; 2008
	*Echinochloa colona*	Argentina; NSW, QLD, WA, Australia; CA, United States	2007–2010
	*Eleusine indica*	Argentina; Bolivia; China; Colombia; Costa Rica; Malaysia; MS, TN, United States	1997; 2006–2007; 2010–2012; 2014
	*Leptochloa virgate*	Mexico	2010
	*Lolium perenne*	Argentina; Brazil; Chile; Japan; Italy; New Zealand; Portugal; Spain; AR, CA, LA, MS, NC, OR, TN, United States	2001–2012; 2014
	*Lolium rigidum*	NSW, VIC, SA, WA, Australia; France; Israel; Italy; South Africa; Spain; CA, United States	1996–1999; 2001; 2003; 2005–2008; 2010; 2013
	*Poa annua*	CA, MO, TN, United States	2010–2011; 2013
	*Sorghum halepense*	Argentina; AR, LA, MS, United States	2005; 2007–2008; 2010
	*Urochloa panicoides*	NSW, Australia	2008
Rubiaceae	*Hedyotis verticillata*	Malaysia	2014

## 2. Environmental Fate

### 2.1. Soil Interactions

Although glyphosate is typically sprayed onto plant foliage, some amount accumulates in the soil through by-spray or being washed off of plant surfaces during precipitation. Once in the soil, glyphosate tightly sorbs to soil particles [30,46,47,48,49,50,51,52,53,54] due to its high affinity for clay minerals [30,55,56], for soil organic matter [48,57,58,59,60] and especially for soil oxides and hydroxides [51,61,62,63,64]. This high affinity for soil particles limits glyphosate’s mobility in the environment, a property considered to be beneficial, since it makes glyphosate somewhat “environmentally benign” [4]. Phosphate, present in fertilizers, competes with glyphosate for binding sites of soil micelles. Under most conditions, phosphate is preferentially sorbed, the presence of which may remobilize previously-bound glyphosate [54].

**Figure 2:**
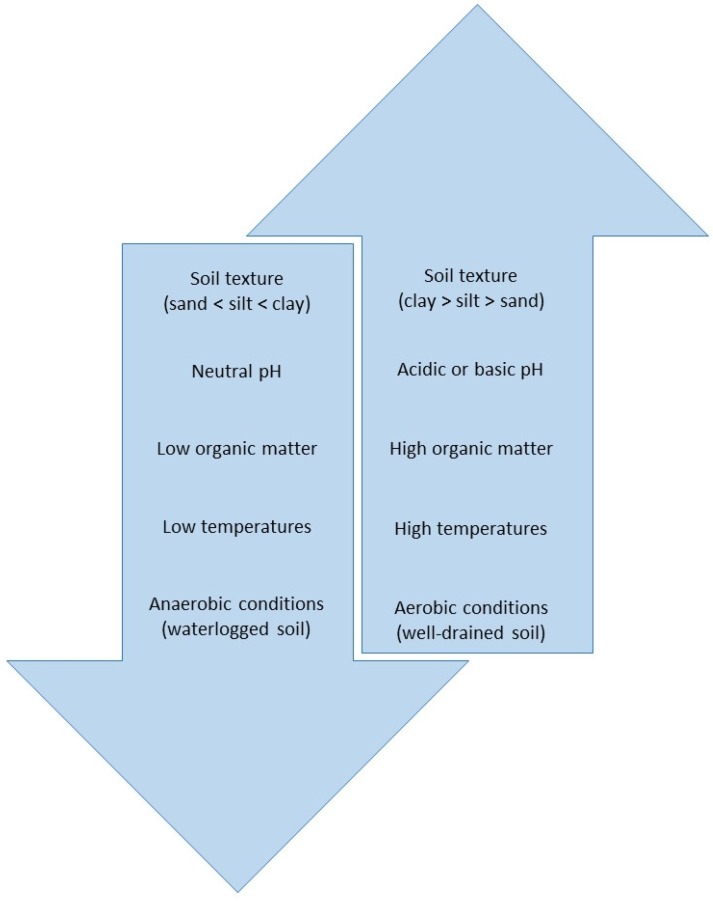
Schematic diagram showing the generalized relationship of environmental factors affecting microbial degradation of glyphosate in soil. Factors listed in the down arrow depress the rate of microbial degradation, while factors listed in the up arrow enhance it.

Glyphosate in soil is degraded by microorganisms [4,52,54]. Microbial degradation occurs via two pathways. The primary pathway produces aminomethylphosphonic acid (AMPA) and glyoxylate. It is worth noting that AMPA, the primary metabolite, is phytotoxic in its own right, negatively affecting plant physiology, although the mechanisms of these effects have not been elucidated [65,66]. In the second pathway, sarcosine and glycine are produced [54]. The degree of soil microbial activity determines the rate of glyphosate degradation. The rate of degradation is also influenced by factors, such as soil texture, pH, organic matter content, temperature and moisture [30,54,67,68,69] (Figure 2). The rate required for 50% dissipation (DT_50_) varies greatly, from 1.2 days–197.3 days. The degradation rates of several studies are summarized in Table 4.

**Table 4:** Summary of glyphosate degradation times in agricultural soils as reported in the literature. DT_50_ refers to the time required for 50% dissipation [4].

Reference	Location	DT_50_ (Days)
Mestdagh, 1979 [70]	France	5–197.3
Mestdagh, 1979 [70]	Sweden	1.2–24.3
Danhaus, 1984 [71]	USA	27.3–55.5
Heinonen-Tanski *et al.*, 1985 [72]	Finland	<58
Ragab *et al.*, 1985 [73]	Canada	<10
Oppenhuizen 1993 [74]	USA	1.7–141.9
Oppenhuizen and Goure, 1993 [75]	Canada	6–21
Thompson *et al.*, 2000 [76]	Canada	10–12
Veiga *et al.*, 2001 [77]	Spain	<30
Simonsen *et al.*, 2008 [78]	Denmark	9

### 2.2. Occurrence in Water

Although glyphosate has rarely been reported in groundwater, when detected, the concentrations are very low. One study conducted by the EPA over six years found glyphosate in seven groundwater samples out of 27,877 samples tested, with a maximum detected concentration of 1.1 µg·L^−1^ [79]. For comparison, the maximum contaminant limit (MCL) for glyphosate is 700 µg·L^−1^ [80].

Glyphosate is conservatively estimated to have an aquatic half-life of 7–14 days in surface waters [4]. Glyphosate is considered to have low potential for runoff due to its high affinity for soils [12]. Contrary to this conventional wisdom, glyphosate has been detected in surface waters, generally within agricultural ditches near the site of application. For example, Edwards *et al.* found glyphosate in all samples for six watersheds in a study conducted over three years sampling runoff following precipitation events. Glyphosate concentrations ranged from 2–94 µg·L^−1^. One sample detected 5153 µg·L^−1^ glyphosate related to an unusually high rate of field application (8.96 kg·ha^−1^, compared to 1.12 and 3.36 kg·ha^−1^ at other sites). This sample is the greatest concentration in runoff found in the literature [81].

The Danish government conducts long-term monitoring of a variety of pesticides, including glyphosate. In a recent report from the project, Kjaer *et al.* found that among the four sites treated with glyphosate, water in adjacent drainage ditches contained glyphosate ranging from less than 0.01–4.7 µg·L^−1^ [82]. This maximum concentration is nearly five-times greater than the MCL for glyphosate in the European Union of 0.1 µg·L^−1^ [83].

Battaglin *et al.* sampled 51 streams in the Midwestern United States at different points in the growing season in 2002. Glyphosate was detected in 36% of 154 samples, depending on timing during the growing season; the concentrations ranged from 0.1–8.7 µg·L^−1^ [84].

A watershed study was conducted by Coupe *et al.* in three watersheds in the Midwestern United States and one watershed in France. In 209 samples collected from three sites in a Mississippi watershed in 2007 and 2008, glyphosate was detected in all samples collected, with concentrations ranging from 0.03–73 µg·L^−1^. In the Iowa watershed, 182 samples were collected with 29% of those containing detectable levels of glyphosate. Two sites sampled in an Indiana watershed showed glyphosate in 100% of 37 samples. Concentrations ranged from 0.07–430 µg·L^−1^. The watershed in France had glyphosate detected in 99.7% of 303 samples. The concentrations ranged from the threshold of detectable limits (0.1 µg·L^−1^) to 86 µg·L^−1^ [85].

Shipitalo and Owens examined glyphosate in runoff from fields with different tillage practices and with different crops. Over a three-year period, a total of 1015 runoff events were sampled in seven watersheds. During that period, one rainfall event resulted in a maximum glyphosate concentration of 887 µg·L^−1^, exceeding the U.S. MCL of 700 µg·L^−1^. Increased instances of glyphosate in runoff were associated with conservation tillage (no-till) as compared to disking or chiseling, while no differences were found between fields planted with corn and soybeans [83].

Based on the above discussion, there can be no doubt that glyphosate runs off of fields where it is applied and into receiving surface waters. Glyphosate concentrations in runoff ranged from 0.01–5153 µg·L^−1^. In many cases, the concentrations of glyphosate detected exceeded the MCL for the U.S. (700 µg·L^−1^) and for the European Union (0.1 µg·L^−1^).

### 2.3. Implications for Non-Target Vegetation

The previously-discussed studies clearly demonstrated that glyphosate may run off from fields where it is applied via soil surface runoff, exposing roots of non-target plants found in agricultural ditches. This exposure pathway is among the least studied for non-target plants. Other exposure pathways are well-studied and include by-spray and drift [86,87,88,89,90,91,92]. Non-target ditch plants are significant in that they contribute to ecosystem services, including sediment trapping, transformation of contaminants and providing habitat for plants and animals.

Following glyphosate’s infiltration into the soil, the roots of non-target plants may be exposed to glyphosate. Only a few studies exist that have investigated the effects of root-zone glyphosate exposure; however, these studies mostly have been carried out in crop species, including beets (*Beta vulgaris*), barley (*Hordeum vulgare*), cotton (*Gossypium hirsutum*), maize (*Zea mays*) and rapeseed (*Brassica napus*) [13,15,16,17,18,19]. To date, limited studies have been published on the effects of root-zone glyphosate exposure on three non-target species, smartweed (*Polygonum hydropiperoides*), maidencane (*Panicum hemitomon*) and creeping water primrose (*Ludwigia peploides*) [93,94,95]. The effects of these studies on various experimental endpoints are summarized in Table 5. Based on a survey of these existing studies, additional investigations into the effects of root-absorbed glyphosate on non-target plants would make major contributions to the literature.

In the field, vegetated agricultural drainage ditches are the primary intercepts for agrochemicals and have also been recently studied for their potential to mitigate pollutants.

Moore *et al.* found that an agricultural drainage ditch dominated by *Polygonum amphibium*, *Leersia oryzoides* and *Sporobolus* sp. was effective at removing the herbicide atrazine and pesticide lambda-cyhalothrin from water during a simulated rainfall event in an edge-of-field ditch. Forty-two to 77% of total measured atrazine was associated with plant material in the ditch, while 61%–93% of measured lambda-cyhalothrin was associated with plant material [96].

**Table 5:** Summary of studies investigating the effects of root-zone glyphosate exposure.

Species	Endpoint	Summary of Effects	Reference
Beet	Betacyanin efflux	Betacyanin efflux increased with increasing glyphosate concentration and time, demonstrating increased cell membrane permeability of root tissue	Fletcher *et al.*, 1980 [15]
(*Beta vulgaris*)			
Barley	Changes in dry weight	23% reduction in shoot dry weight	Penn and Lynch, 1982 [16]
(*Hordeum vulgare*)			
Cotton	Changes in fresh weight; lateral root development	50% reduction in fresh weight of cotyledons, hypocotyls and roots; inhibition of lateral root development	Pline *et al.*, 2002 [17]
(*Gossypium hirsutum*)			
Maize	Changes in fresh weight; visual symptoms	Growth reduction of up to 44% of fresh weights following a logistic response curve; hormesis effect noted for exposures of less than 1 µg·L^−1^; wilting and chlorosis for exposures greater than 1 µg·L^−1^	Wagner *et al.*, 2003 [13]
(*Zea mays*)			
Maize	Changes in fresh weight	Growth reduction of 50% of fresh weights for exposures of 30 mg·L^−1^	Alister *et al.*, 2005 [18]
(*Zea mays*)			
Rapeseed	Changes in dry weight; visual symptoms	Growth reduction of 83% of dry weights for roots and 43% reduction for shoots; leaf chlorosis and necrosis for exposures of 20 µM·L^−1^ or greater	Petersen *et al.*, 2007 [19]
(*Brassica napus*)			
Smartweed	Changes in leaf chlorophyll content and dry weight; survival	Dose-dependent reductions in leaf chlorophyll content in *P. hydropiperoides* and *P. hemitomon*; no differences in dry weight for either species; survival at 10 µg·L^−1^ for *P. hydropiperoides* and mortality at higher concentrations; survival by *P. hemitomon* except at 10,000 µg·L^−1^	Saunders *et al.*, 2013 [93]
(*Polygonum hydropiperoides*)			
Maidencane			
(*Panicum hemitomon*)			
Smartweed	Changes in leaf chlorophyll content, chlorophyll fluorescence parameters, and dry weight	Reduction in chlorophyll content for treated *P. hydropiperoides*; species-specific reductions in chlorophyll fluorescence parameters; no differences in dry weight	Saunders and Pezeshki, 2014 [94]
(*Polygonum hydropiperoides*)			
Creeping water primrose			
(*Ludwigia peploides*)			
Creeping water primrose	Changes in morphology and dry weight	Hormesis effect depending on root density of connected ramets	Saunders and Pezeshki, 2015 [95]
(*Ludwigia peploides*)			

Cooper *et al.* investigated the potential of three agricultural ditches dominated by *Polygonum* sp., *Leersia* sp. and *Ludwigia* sp. to remove atrazine, lambda-cyhalothrin and the pesticide bifenthrin and found that 57%–99% of the measured pesticides were associated with the ditch vegetation plant material [97]. In another study, Cooper *et al.* found that three ditch species, *Ludwigia peploides*, *Polygonum amphibium* and *Leersia oryzoides*, were effective at the removal of the insecticide pyrethroid esfenvalerate [98]. Bouldin *et al.* found that unvegetated microcosms had higher concentrations of atrazine and lambda-cyhalothrin as compared to vegetated microcosms, with *Ludwigia peploides* and *Juncus effusus* removing significant amounts of the agrochemicals from the water column [99]. Bouldin *et al.* further found that the ditch species *Ludwigia peploides* and *Juncus effusus* were successful in removing atrazine and lambda-cyhalothrin from hydroponic solutions containing simulated runoff [100].

Krӧger *et al.* investigated the effects of hydraulic residence time in ditches on the removal of nutrients and found that ditches can remove up to 94% of dissolved inorganic phosphate, 96% of nitrate and 85% of ammonium [101]. Stehle *et al.* recently reviewed this topic and conducted a meta-analysis of 24 publications regarding vegetated treatment systems, such as agricultural ditches and buffer strips, and found that more than half of the studies reported removal of agrochemicals that exceeded 70% [102].

Saunders *et al.* found that root-zone glyphosate exposure in two ditch species, *Polygonum* *hydropiperoides* and *Panicum hemitomon*, led to dose-dependent reductions in leaf chlorophyll content, while biomass was unaffected. *P. hydropiperoides* was more sensitive to lower concentrations of root-zone glyphosate exposure as compared to *P. hemitomon*, with *P. hydropiperoides* displaying greater mortality at lower exposure concentrations than *P. hemitomon* [93].

**Figure 3:**
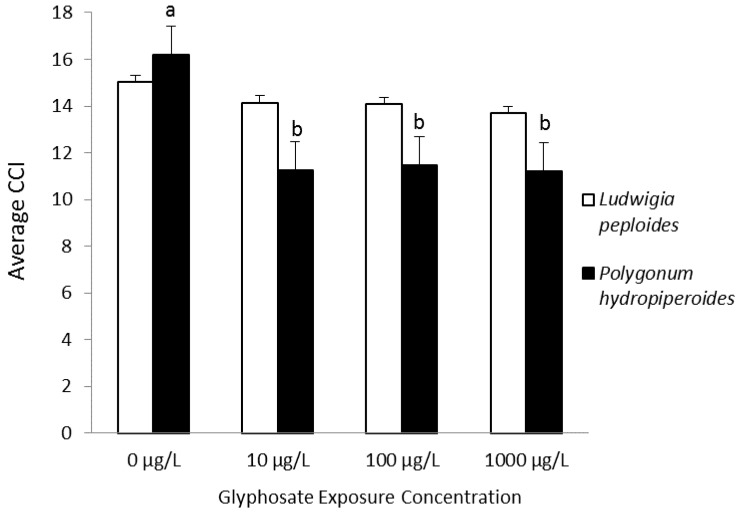
Average chlorophyll content index (CCI) measurements for Days 1–17. Bars represent the means ± the standard error. Lowercase letters (a,b) refer to significant differences across treatments within species. Differences considered significant at α < 0.05 [94], Copyright 2014, Wiley.

Saunders and Pezeshki investigated the physiological effects of a range of environmentally-relevant root-zone glyphosate exposure concentrations in two species commonly found in agricultural ditches. Both species displayed transient reductions in chlorophyll fluorescence parameters, Fv/Fm (ratio of variable fluorescence to maximal fluorescence) and yield. Leaf chlorophyll content was reduced over the observation period in treated plants of *Polygonum hydropiperoides*, while *Ludwigia peploides* was unaffected, as shown in Figure 3. Biomass was unaffected for either species. The photoinhibition demonstrated by reductions in chlorophyll fluorescence parameters was not sufficient to affect *L. peploides* leaf chlorophyll content [94].

Saunders and Pezeshki studied the effects of physiological integration and spatial heterogeneity of root-zone glyphosate exposure in connected ramets of *Ludwigia peploides*. Glyphosate exposure in the root-zone affected plants differently depending on the root density of exposed ramets. For all connected ramet pairs, mother ramets had three-times greater root densities than daughter ramets, leading to the designations “high root density” when referring to mother ramets and “low root density” when referring to daughter ramets. When high root density mother ramets were exposed to root-zone glyphosate, plants had identical morphology to untreated controls, with mother ramets having greater numbers of leaves and shoots as compared to daughter ramets. When low root density daughter ramets were exposed to root-zone glyphosate, plants displayed an opposite morphology, interpreted as a hormesis effect, with daughter ramets having greater numbers of leaves and shoots compared to mother ramets. In plants in which the high root density mother ramets were exposed to root-zone glyphosate, glyphosate was sequestered in the metabolic sinks of high root density where the glyphosate was applied. In plants in which the low root density daughter ramets were exposed to root-zone glyphosate, glyphosate traveled throughout the plant, moving away from the low density roots where the glyphosate was applied and toward the metabolic sinks of the mother ramet high density roots; these plants exhibited a hormesis effect, in which growth was greater for daughter ramet leaves and shoots as compared to mother ramets [95].

## 3. Conclusions

Based on the literature presented, the following points may be recapitulated: (1) glyphosate often runs off of fields where it is applied; (2) glyphosate can be translocated by plant roots; and (3) glyphosate can affect plant functioning in non-target plants found in agricultural ditches. These findings have practical applications. For example, the information may be utilized by government agencies or land managers seeking to understand the effects of glyphosate runoff on the vegetation in the receiving agricultural ditches. By employing best management practices, such as vegetated buffer strips composed of species found to be tolerant of glyphosate runoff, land managers can reduce the amount of glyphosate transported downstream from farms and minimize additional unintended consequences of intensive use of this broad-spectrum herbicide. In addition, these findings promote an increased awareness that adverse effects of glyphosate runoff on plants do in fact exist, contrary to conventional wisdom.

Transitioning away from glyphosate and glyphosate-resistant cropping systems is unlikely in the future. The most recent statistics for the United States show that, in 2014, 94% of soybeans, 91% of cotton and 89% of corn acreage was planted with herbicide-tolerant crop varieties. Those herbicide-tolerant crops may receive herbicides other than glyphosate, but glyphosate-resistance was the first among herbicide-tolerant crop technology. Furthermore, developing countries are investing in the glyphosate-resistant cropping system, increasing the use of glyphosate in new areas. A host of concerns regarding glyphosate use exist, the most relevant of which is increased selection pressure by glyphosate to shift weed populations or produce glyphosate-resistant weeds. These are important environmental and societal issues for which the costs and benefits of such widespread use of glyphosate should be assessed.
